# Distinct Roles of Acidophiles in Complete Oxidation of High-Sulfur Ferric Leach Product of Zinc Sulfide Concentrate

**DOI:** 10.3390/microorganisms8030386

**Published:** 2020-03-10

**Authors:** Maxim Muravyov, Anna Panyushkina

**Affiliations:** Winogradsky Institute of Microbiology, Research Centre «Fundamentals of Biotechnology» of the Russian Academy of Sciences, Leninsky Ave., 33, bld. 2, 119071 Moscow, Russia; zhuravleva-inmi@mail.ru

**Keywords:** biooxidation, bioleaching, two-step process, zinc concentrate, acidophilic microorganisms

## Abstract

A two-step process, which involved ferric leaching with biologically generated solution and subsequent biooxidation with the microbial community, has been previously proposed for the processing of low-grade zinc sulfide concentrates. In this study, we carried out the process of complete biological oxidation of the product of ferric leaching of the zinc concentrate, which contained 9% of sphalerite, 5% of chalcopyrite, and 29.7% of elemental sulfur. After 21 days of biooxidation at 40 °C, sphalerite and chalcopyrite oxidation reached 99 and 69%, respectively, while the level of elemental sulfur oxidation was 97%. The biooxidation residue could be considered a waste product that is inert under aerobic conditions. The results of this study showed that zinc sulfide concentrate processing using a two-step treatment is efficient and promising. The microbial community, which developed during biooxidation, was dominated by *Acidithiobacillus caldus*, *Leptospirillum ferriphilum, Ferroplasma acidiphilum, Sulfobacillus thermotolerans, S. thermosulfidooxidans,* and *Cuniculiplasma* sp. At the same time, *F. acidiphilum* and *A. caldus* played crucial roles in the oxidation of sulfide minerals and elemental sulfur, respectively. The addition of *L. ferriphilum* to *A. caldus* during biooxidation of the ferric leach product proved to inhibit elemental sulfur oxidation.

## 1. Introduction

At present, zinc is one of the main non-ferrous metals and is currently the fourth most widely consumed metal after iron, aluminum, and copper [1]. The main consumers of zinc are steel galvanization, alloy (brass and bronze) production, and the chemical industry [2]. Annually, the worldwide production of zinc exceeds 13 million tons [3]. Currently, zinc sulfide ores are the main source of zinc metal production and more than 85% of the world’s zinc is produced from zinc sulfide concentrates using the roast–leach–electrowinning process [1,4]. To date, sulfide ore deposits of the Ural region (Sverdlovsk, Chelyabinsk, and Orenburg regions and Bashkortostan Republic) supply more than 90% of the zinc production in Russia. Sphalerite, chalcopyrite, pyrite, and pyrrhotite are major sulfide minerals composing typical copper-zinc ores in the Urals. Many polymetallic ores are characterized by a thin impregnation of sulfide minerals and mutual intergrowth, which complicates the production of high-grade monometallic concentrates and increases the production cost. Due to this, low-grade monometallic sulfide concentrates (zinc concentrates with relatively high copper content and copper concentrates with relatively high zinc content) are produced at numerous concentrators. This results in the losses of non-ferrous metals in slag during the pyrometallurgical processing of sulfide concentrates and creates technological difficulties at various stages of the metal production process. Technologies based on biooxidation of sulfides may be promising for the processing of the low-grade concentrates of non-ferrous metals, including polymetallic ones.

Biooxidation of sulfide sources based on the activity of acidophilic chemolithotrophic microorganisms is used for decades for the recovery of metals [5,6]. Biohydrometallurgy has many advantages over conventional chemical hydrometallurgy and pyrometallurgy, which include (i) lower cost, (ii) low environmental impact, (iii) generation of less hazardous waste, and (iv) no need for toxic chemicals and high energy [7]. For instance, the environmental benefits of the operations based on the use of acidophilic microorganisms for processing of copper-zinc concentrates have been shown [8,9].

Metal leaching from sulfides at elevated temperatures using ferric sulfate solutions generated during biooxidation with acidophilic microorganisms can be promising for processing of the low-grade zinc concentrates. Leaching of sphalerite with ferric sulfate in acidic media is generally slow and incomplete due to the formation of passivating layers such as elemental sulfur [10,11]. The rate of sphalerite oxidation naturally decreases with an increase in the duration of the process. Particularly, this is due to an increase in the number of reaction products that prevent the oxidizing agent from accessing sulfide particles and, therefore, complete zinc extraction into the liquid phase becomes a difficult task [12].

A biohydrometallurgical process based on the combination of ferric leaching of the concentrate and subsequent complete oxidation of leach products (with a high content of elemental sulfur in the solid phase) by acidophilic microorganisms can be a suitable method for complete recovery of metals from the low-grade zinc concentrates. This approach will make it possible to extract metals from the sulfide concentrate and to obtain the leach residue, which is an inert product that is suitable for dump storage.

Communities of acidophilic chemolithotrophic microorganisms functioning in industrial processes of biooxidation of sulfide raw materials (for instance, BIOX^TM^, BIONORD^®^, and BIOCOP^TM^) are phylogenetically diverse [13]. The predominance of certain species is determined by various environmental parameters, such as temperature, pH, pulp density, metal concentration, the concentration of dissolved gases (oxygen and carbon dioxide), and the presence of dissolved organic substances. Some members of these microbial communities are able to oxidize ferrous iron (for instance, *Leptospirillum* spp. and *Ferroplasma acidiphilum*), while other microorganisms can oxidize reduced sulfur compounds (such as *Acidithiobacillus caldus* and *A. thiooxidans*) or possess both oxidizing abilities (*A. ferrooxidans*, *Sulfobacillus* spp., and others) [14].

The goal of this research was to study biooxidation of the products of ferric leaching of zinc sulfide concentrate by the community of acidophilic chemolithotrophic microorganisms, as well as to determine the role of its members in biooxidation. The solid leach product contained large amounts of elemental sulfur. This study is an important milestone in the development of the second (biological) step of processing of low-grade zinc sulfide concentrates.

## 2. Materials and Methods

### 2.1. Materials

The products of ferric leaching of the concentrate of zinc sulfide ore of the Tarnier deposit (Sverdlovsk region, Russia) were used in experiments. The conditions and procedure for the four-cycle ferric leaching of the concentrate were previously described [12]: 80 °C, pH 1.3, 10% (*w*/*v*) pulp density, and 25 g/L initial ferric iron. A ferric iron-containing biosolution that was used for leaching was obtained using acidophilic microorganisms. The particle size distribution of the concentrate sample had a P_90_ of 44 μm. Leach residue and solution were the leach products. Table 1 shows the chemical and mineralogical composition of the zinc concentrate and the leach residue obtained after leaching of the concentrate. The leach solution contained the following (g/L): Fe^2+^, 24.2; Fe^3+^, 1.12; Zn^2+^, 10.0; Cu^2+^, 0.3. The pH value of the solution was 1.25.

### 2.2. Biooxidation Experiments

Two series of experiments on the biooxidation of the products of ferric leaching by the microbial community were carried out under batch conditions at 40 °C. In the first series of the experiments, a bioreactor (2.0 L) contained 100 g of the leach residue, 900 mL of the mixture of the leach solution and iron-free 9K medium [15] in a 1:1 ratio, 0.005 wt.% yeast extract as a source of organic nutrients, and 100 mL of inoculum. The microbial community, which was developed during ferric iron bioregeneration for ferric leaching of the zinc concentrate, was used as an inoculum. The community consisted of the following microorganisms: *L. ferriphilum, S. thermosulfidooxidans, S. thermotolerans, A. caldus, A. thiooxidans, F. acidiphilum, Acidiplasma* sp., and *Cuniculiplasma* sp. [16]. The experiments were carried out with aeration of 4 L/min and at a stirring speed of 500 rpm. The initial pH value was 1.5. The second series of biooxidation experiments was carried out with pure microbial cultures and their combinations. The leach residue (1 g), leach solution and iron-free 9K medium in a ratio of 1:1 (90 mL), yeast extract (0.005 wt.%), and inoculum (10 mL) were added to 250 mL conical flasks. The initial ambient pH value was adjusted to 1.8 with 5 M H_2_SO_4_. The flasks were incubated on Unimax-1010 rotor shakers (Heidolph Instruments, Schwabach, Germany; 190 rpm) in Inkubator-1000 thermostats (Heidolph Instruments, Schwabach, Germany) at 40 °C. The pure cultures that were used in experiments were as follows: *L. ferriphilum* MP1, *S. thermosulfidooxidans* MP2, and *A. caldus* MP3 (isolated and identified during this study) and the type strain *F. acidiphilum* Y^T^ obtained from the Collection of Microorganisms of Winogradsky Institute of Microbiology, Russian Academy of Sciences (Moscow, Russia). *F. acidiphilum* Y^T^ that was used in experiments was preliminarily cultured in the salt base of the 9K medium supplemented with ferrous iron (3.5 g/L) and yeast extract (0.02 wt.%). The flask and bioreactor experiments were carried out in triplicate and duplicate, respectively.

### 2.3. Analysis of Microbial Community Structure

#### 2.3.1. 16S rRNA V3-V4 Amplicon Metabarcoding

The microbial community structure was studied by metabarcoding analysis with specific molecular markers (V3-V4 hypervariable region of the 16S rRNA gene). Biomass for metabarcoding was obtained by centrifugation. First, a native suspension was centrifuged to precipitate solids (100 *g*, 2 min) and cell pellets were further collected by centrifugation of the obtained supernatant (5000 *g*, 15 min). After that, the biomass was sequentially washed by centrifugation (10,000 *g*, 5 min) with the iron-free 9K medium [15], pH 1.5, and with the same medium with a neutral pH. Cell pellets were resuspended in 300 μL of the lysis buffer (0.15 M NaCl; 0.1 M Na_2_-EDTA; 15 mg/mL lysozyme; pH 8.0). DNA was extracted from the biomass using the PowerSoil® Isolation Kit (MO BIO Laboratories Inc., Carlsbad, CA, USA) according to the manufacturer’s recommendations. Qualitative and quantitative assessment of the obtained DNA preparations was carried out with a DropSense-96® spectrophotometer (Trinean, Ghent, Belgium). The temperature–time profile of the PCR was as follows: 30 cycles, denaturation at 95 °C for 15 s, primer annealing at 58 °C for 15 s, and DNA elongation at 72 °C for 25 s; final DNA elongation, 72 °C, 5 min. The Pro341F (5’-CCTACGGGNBGCASCAG-3’) and Pro805R (5’-GACTACNVGGGTATCTAATCC-3’) universal primers were used. For subsequent sequencing, the amplicons were separated by agarose gel electrophoresis, excised from the gel, and purified using the Cleanup Standard kit for the purification of DNA from agarose gels and reaction mixtures (Evrogen, Moscow, Russia). Multiplex amplicon libraries for sequencing on the Illumina MiSeq platform (Illumina, San Diego, CA, USA) were prepared using the NEBNext® kit for fragment libraries (New England BioLabs, Ipswich, MA, USA). Sequencing was performed using the reagent kit under the conditions, providing for a reading length of 300 nucleotides from each end of the amplicon. Demultiplexing was performed as previously described [17]. After demultiplexing, all reads were subjected to a stringent sequence quality filtering process and reads containing sequences of the 16S rRNA primers were filtered out using the CLC Genomics Workbench version 10.0 (Qiagen, Redwood City, CA, USA). The QIIME open-source software pipeline [18] and the Silva132 database [19] were used to choose operational taxonomic units (OTU) and to perform the taxon-based assignment; all parameters were set to default values. Approximately 10,000 fragments with an average length of 486 nucleotides were analyzed.

#### 2.3.2. Isolation of Pure Cultures and Taxonomic Research

Pure cultures of autotrophic and mixotrophic microorganisms were isolated from the pulp liquid phase by the method of serial terminal tenfold dilutions. The modified 9K medium containing ferrous sulfate [20] as an energy source and the same 9K medium supplemented with yeast extract (0.02 wt.%) were used to isolate the autotrophic and mixotrophic iron oxidizers, respectively. Similarly, the 9K medium containing elemental sulfur (10 g/L) instead of ferrous iron and the same medium supplemented with 0.02 wt.% yeast extract were used to isolate sulfur-oxidizing microorganisms. Isolation of pure cultures was carried out in 250 mL Erlenmeyer flasks containing 100 mL of the medium and 10% (*v*/*v*) of the inoculum. The flasks were incubated on Unimax-1010 rotor shakers (Heidolph Instruments, Schwabach, Germany; 170 rpm) in Inkubator-1000 thermostats (Heidolph Instruments, Schwabach, Germany) at 40 °C. The DNA extraction and purification, PCR amplification of the 16S rRNA gene fragments, amplicon purification and subsequent sequencing, as well as phylogenetic identification of the pure microbial cultures were carried out as previously described [21].

### 2.4. Analytical Methods

Quantitative assessment of microorganisms was carried out by direct counts in the Goryaev chamber and by the method of tenfold dilutions using an Amplival (Carl Zeiss, Jena, Germany) microscope equipped with a phase contrast device. Microscopy was also used to assess morphological diversity and the physiological state of the cultures. The рН value was measured with a рН-150МI pH meter (Izmeritelnaya tekhnika, Moscow, Russia). The concentrations of the ferric iron in the aqueous phase were determined from the reaction with potassium thiocyanate [22] using a PE-5400UF spectrophotometer (ECROSKHIM, St. Petersburg, Russia) at λ = 480 nm. The total iron concentration was determined by the same method after the reaction of the sample with ammonium persulfate. The ferrous ion concentration was defined as the difference between the second and the first values. The concentrations of copper and zinc were determined on the Perkin Elmer 3100 flame atomization atomic absorption spectrometer (PerkinElmer, Waltham, MA, USA).

Solids (original concentrate and leach residues) were analyzed by a wet chemical method using a 1:3 ratio of concentrated nitric acid and concentrated hydrochloric acid at the boiling temperature of the mixture, followed by flame atomization atomic absorption spectroscopy (AAnalyst-800, PerkinElmer, Waltham, MA, USA) for iron, copper, and zinc. Gravimetric analysis with barium chloride was used for sulfur [23]. The elemental sulfur content was determined using its dissolution in carbon tetrachloride at 50 °C, followed by gravimetric analysis. The content of sulfate sulfur was determined by gravimetric analysis with barium chloride after the reaction with sodium carbonate at the boiling temperature [24]. The mineralogical composition of the concentrate and of the leach residues were determined by X-ray diffraction on a DRON-2 diffractometer (Burevestnik, St. Petersburg, Russia). The sulfidic mineral contents were adjusted according to the element contents that were determined by the phase methods.

## 3. Results and Discussion

Earlier, leaching of the low-grade zinc sulfide concentrate with a ferric sulfate solution generated using an autotrophic microbial community made it possible to recover 92.3 wt.% of zinc and 51.6 wt.% of copper after 14.25 h of the process. The residue yield was 54 wt.%. As a result, a leach residue with a high content of elemental sulfur (see Table 1) and a leach solution were formed [12]. In this study, we proposed biooxidation of these leach products by a community of acidophilic chemolithotrophic microorganisms to increase the level of metal recovery from the concentrate and to produce relatively inert waste with a minimum content of toxic elements. The main chemical transformations during the proposed process can be characterized by simplified reaction equations:

ZnS + 2Fe^3+^ = Zn^2+^ + S^0^ + 2Fe^2+^, (1)

CuFeS_2_ + 4Fe^3+^ = Cu^2+^ + 2S^0^ + 5Fe^2+^, (2)

4Fe^2+^ + 4H^+^ + O_2_ = 4Fe^3+^ + 2H_2_O, (3)

2S^0^ + 2H_2_O + 3O_2_ = 2H_2_SO_4_, (4)

3Fe_2_(SO_4_)_3_ + 12H_2_O + M_2_SO_4_= 2MFe_3_(SO_4_)_2_(OH)_6_↓ + 6H_2_SO_4_ (5)

where M = K^+^, NH_4_^+^, or H_3_O^+^.

According to these reactions, iron oxidation is accompanied by the consumption of protons, while the oxidation of sulfur results in the generation of acid. Therefore, co-oxidation of sulfur and iron can reduce the precipitation of the oxidant of sulfide minerals according to reaction (5), the intensity of which increases with an increase in pH [25].

In the previous study, the microbial community growing at 40 °C was shown to have an advantage in terms of ferric iron bioregeneration during the joint oxidation of the residue (1% (*w*/*v*)) and the leach solution over the communities cultured at 35 and 45 °C [16]. Furthermore, ferrous iron and elemental sulfur were oxidized first, while sulfide oxidation was negligible [16]. The goal of this research was to achieve complete oxidation of sulfides and elemental sulfur in the ferric leach residue, and the duration of the process was, therefore, increased.

### 3.1. Oxidation by Microbial Community in Bioreactor

**Figure 1:**
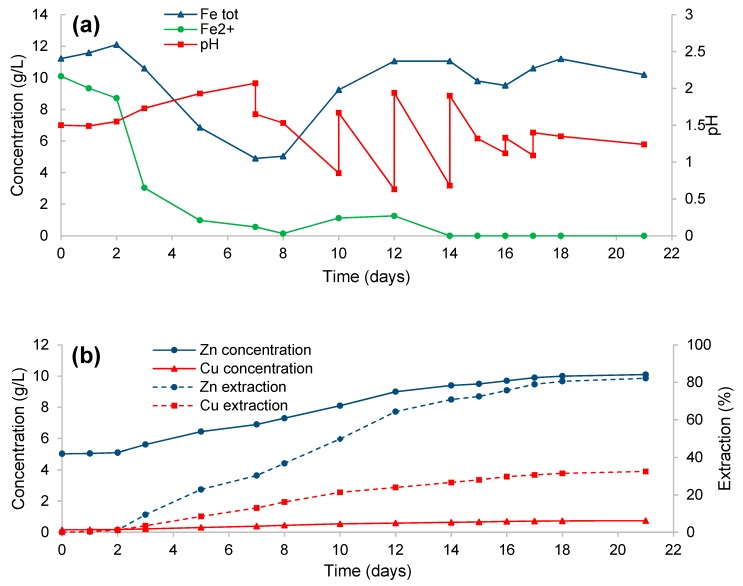
Changes in the physicochemical parameters of the liquid phase during biooxidation of the ferric leach products in the bioreactor: (**a**) total iron concentration, ferrous iron concentration, and the pH value; (**b**) zinc and copper concentrations and extraction levels.

The experiments were carried out with a pulp density of 10% (*w*/*v*). The pH of the pulp was monitored, and calcium carbonate or sulfuric acid was added when necessary to maintain the pH value in the range of 0.6–2.0. The main physicochemical conditions of the aqueous phase of the pulp during biooxidation of the ferric leach residue are shown in Figure 1. The highest rate of the decrease in the ferrous iron concentration was observed from the second day to the fifth day of the process. At the same time, the pH value increased from 1.55 to 1.93, despite jarosite precipitation, which was characterized by a decrease in the concentration of total iron in the medium. Probably, the rate of elemental sulfur oxidation by microorganisms was low during this period. The concentration of ferrous iron decreased to 0.14 g/L on the eighth day of the process. After that, it increased to 1.26 g/L for a relatively short time. This increase in ferrous iron concentration could be associated with inhibition of iron-oxidizing microorganisms due to low pH values (0.6–0.9 units) during this period [26,27,28]. Complete ferrous iron oxidation was observed on day 14. Starting from the eighth day of the process, the rate of elemental sulfur oxidation increased, resulting in a rapid decrease in the ambient pH value. Therefore, calcium carbonate was added on days 10, 12, 14, 16, and 17 (to precipitate sulfate ion, in particular). The total consumption of calcium carbonate was 0.99 g/g ferric leach residue. It is noteworthy that intense oxidation of elemental sulfur coincided with almost complete oxidation of ferrous iron in the medium. Zinc concentration characterizing sphalerite oxidation significantly increased from the second day to the 18th day of the process. Copper concentration, which is indicative of chalcopyrite oxidation, monotonously increased throughout the entire process and reached 0.742 g/L. Microbial abundance reached 10^10^ cells/mL during the biooxidation process.

**Figure 2:**
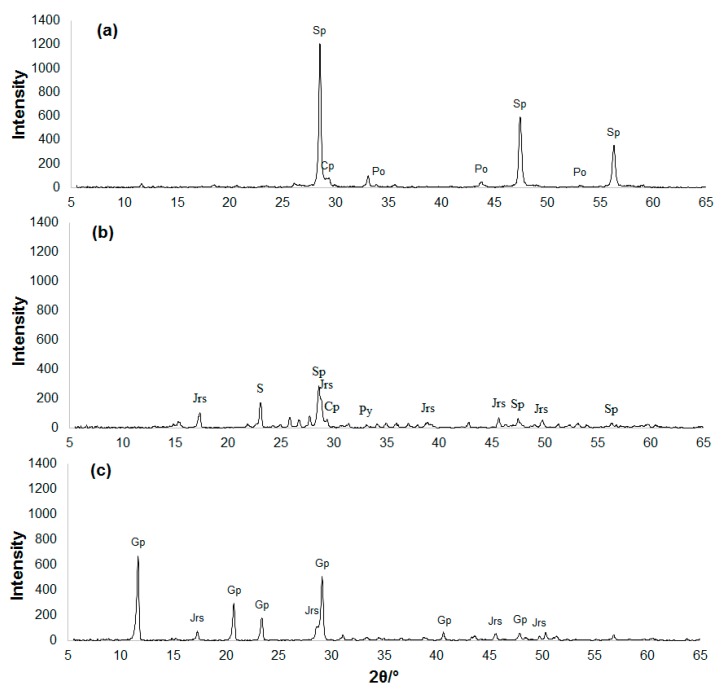
X-ray diffraction patterns of the zinc concentrate (**a**), ferric leach residue (**b**), and the residue after biooxidation by the microbial community in the bioreactor (**c**) (Sp, sphalerite; Po, pyrrhotite; Cp, chalcopyrite; Jrs, jarosite; Gp, gypsum; S, elemental sulfur).

**Figure 3:**
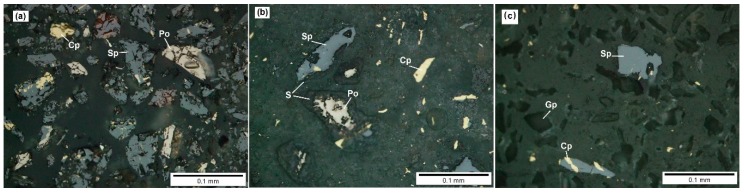
Micrographs of polished sections of the zinc concentrate (**a**), ferric leach residue (**b**), and the residue after biooxidation by the microbial community in the bioreactor (**c**) (Sp, sphalerite; Po, pyrrhotite; Cp, chalcopyrite; Gp, gypsum; S, elemental sulfur).

The residue obtained after the process of joint biooxidation of the ferric leach products of the zinc concentrate was studied. Figure 2 shows the X-ray diffraction pattern of the mineralogical composition of the residue in comparison with the X-ray diffraction patterns of the original zinc concentrate and ferric leach residue. A decrease in the intensity of the peaks for sphalerite in the ferric leach residue (compared to the original concentrate) and their almost total absence in the biooxidation residue can be seen. In general, the biooxidation residue consisted of two main phases: jarosite and gypsum. Figure 3 shows micrographs of polished sections of the zinc concentrate (Figure 3a), its ferric leach residue (Figure 3b), and biooxidation residue (Figure 3c). Gypsum prevailed in the biooxidation residue; sphalerite and chalcopyrite particles were identified (Figure 3c). The peaks for sphalerite and chalcopyrite were not detected in the X-ray diffraction pattern of the biooxidation residue (Figure 2c) due to small quantities of these minerals. The micrographs indicate that the particles of sphalerite and pyrrhotite in the ferric leach residue are coated with a layer of the oxidation reaction product (elemental sulfur), while this layer is absent in the biooxidation residue due to the activity of sulfur-oxidizing microorganisms. Therefore, the role of microorganisms during biooxidation was not only the generation of the oxidizing agent, but also the purification of the sulfidic surface from reaction products. The presence of such products causes passivation of the surface of sulfides and significantly slows down the oxidation process [10].

The data on the content of the main elements and minerals in the biooxidation residue and the levels of oxidation of sulfide minerals and elemental sulfur, calculated on their basis, made it possible to assess the efficiency of biooxidation of the ferric leach residue. The results are shown in Table 2 and Table 3. According to these data, the biooxidation residue contained only 0.52 wt.% of zinc, 0.55 wt.% of copper, and 0.40 wt.% of elemental sulfur, which was caused not only by the oxidation of sulfides, but also by the increased residue yield (212 wt.%) due to the precipitation of jarosite and gypsum. These contents of metals are characteristic of the wastes of ore concentration, including the tailings of flotation of sulfide ores [29]. Minerals that were inert under aerobic conditions (jarosite and gypsum) composed 97 wt.% of the biooxidation residue, which can be considered dump waste. The final oxidation levels (compared to the original concentrate) of pyrrhotite and sphalerite reached the highest values among sulfide minerals since these minerals have low rest potentials in comparison with chalcopyrite. As a result, they are the first minerals that are oxidized during their galvanic interaction [30,31,32]. Chalcopyrite is normally highly refractory to biooxidation in acidic media containing ferric iron. To overcome this problem, a substantial amount of research has been carried out in recent decades. These studies included the use of thermophilic microorganisms, maintenance of the ambient redox potential at relatively low values, the use of catalysts (for instance, silver ions), and galvanic interaction of sulfides [33,34,35,36,37]. The oxidation level of elemental sulfur during biooxidation of the ferric leach residue of the zinc concentrate reached high values due to the activity of sulfur-oxidizing microorganisms.

The composition of the microbial community involved in the biooxidation of the products obtained after the step of ferric leaching of the zinc sulfide concentrate was determined by the 16S rRNA metabarcoding analysis (V3–V4 region) and the method of terminal tenfold dilutions used for the isolation of pure cultures of iron- and sulfur-oxidizing microorganisms (Table 4). According to the results of the metabarcoding analysis, bacteria of the genera *Acidithiobacillus*, *Leptospirillum*, and *Sulfobacillus*, as well as archaea of the order *Thermoplasmatales*, prevailed in the biooxidation process. The closest phylogenetic relatives of the predominant bacteria of the genus *Acidithiobacillus* belonged to an autotrophic sulfur-oxidizing species *A. caldus*. According to the data on tenfold dilutions, members of this species were the most abundant bacteria in the community (10^10^ cells/mL). A pure culture of *A. caldus* was isolated from the community using the medium containing sulfur and yeast extract or containing no organic substrates, indicating its capability of efficient growth both in the presence of small amounts of organic compounds and under strictly autotrophic conditions. Mixotrophic bacteria of the genus *Sulfobacillus* oxidizing both ferrous iron and elemental sulfur composed the second most numerous microbial group. The results of the metabarcoding analysis, the method of tenfold dilutions, and microscopy (used to distinguish two *Sulfobacillus* species by different morphology of their cells) revealed two predominant members of this genus. These bacteria were phylogenetically close to *S. thermotolerans* (up to 10^7^ cells/mL) and *S. thermosulfidooxidans* (10^8^ cells/mL) strains. A pure culture of the predominant strain assigned to the species *S. thermosulfidooxidans* was isolated. Iron-oxidizing autotrophic bacteria, isolated into pure culture from the fifth tenfold dilution in the medium containing ferrous iron as a sole source of energy, were assigned to the species *L. ferriphillum*. The iron-oxidizing species *F. acidiphilum* and organoheterotrophic archaeon *Cuniculiplasma* sp. were identified in the community by metabarcoding analysis but were not isolated into pure cultures. Closely related microorganisms are usually not differentiated only on the basis of differences in the V3–V4 region. Nevertheless, it was possible to identify *F. acidiphilum* at the species level due to the high variability of the V3–V4 region within the genus *Ferroplasma*. The species *F. acidiphilum* was also the only species of the genus *Ferroplasma* present in the inoculum, which was used in bioleaching experiments.

Overall, analysis of the 16S rRNA gene sequences made it possible to assign the isolated microorganisms to the following strains: *L. ferriphilum* strain MP1 (MN780596 GenBank; 1516 bp), *S. thermosulfidooxidans* strain MP2 (MN780597 GenBank; 1458 bp), and *A. caldus* strains MP3 and MP4 (MN780598 and MN780599 GenBank, respectively; 1475 bp). The MP4 and MP3 strains were isolated using the same sulfur-containing medium with or without yeast extract, respectively. Their 16S rRNA gene sequences showed 100% identity (100% coverage) and were further considered to belong to the same strain designated MP3.

Such microbial diversity during biooxidation of the ferric leach products was primarily determined by the presence of energy substrates (ferrous iron and elemental sulfur), as well as by the temperature of the process (40 °C) falling within the temperature limits for growth of these microorganisms or even optimal temperature ranges (*S. thermotolerans*, *F. acidiphilum*, and *Cuniculiplasma* sp.) [14,38]. The predominance of mixotrophs and organoheterotrophs was also caused by the presence of microbial metabolites (low molecular weight organic compounds) in the solution of ferric leaching of the zinc concentrate, which were produced at the initial step of the solution generation (for leaching of the concentrate) by the autotrophic microbial consortium. The products of cell lysis, which were released into the solution during high-temperature ferric leaching, were other sources of organic compounds.

Acidophilic heterotrophs can grow on a wide range of amino acids, sugars, small molecular weight organic acids, and alcohols, as well as some polysaccharides. While the association between chemolithotrophs and heterotrophs might be commensal (beneficial only to one partner) in some circumstances, in others it is mutualistic in that removal of accumulating organic materials may “detoxify” the environment for the chemolithotrophs [39]. Archaea of the genus *Cuniculiplasma* were shown to utilize the components of the yeast and beef extract [38], which implicates the possibility of their growth on the microbial lysates as well. Bacteria of the genus *Sulfobacillus* were shown to be able to use yeast extract and a wide range of organic compounds: carbohydrates, organic acids, and amino acids [28,40]. In bacteria of the genus *Sulfobacillus*, organic substances serve as sources of carbon and energy under mixotrophic and heterotrophic conditions [41]. *Sulfobacillus* strains were also shown to be able to utilize glycolic acid (a metabolite of actively growing acidophiles), while members of the genus *Ferroplasma* could use the organic carbon of the products of *A. caldus* and *Leptospirillum* spp. cell lysis [42,43]. Moreover, *S. thermotolerans and S. thermosulfidooxidans* can utilize acetate and propionate that may be accumulated in the medium during the growth of the microbial community [44,45]. The addition of yeast extract to the medium promoted biooxidation due to the development of microorganisms of the genera *Sulfobacillus* and *Ferroplasma* strains [46,47].

### 3.2. Oxidation by Microorganisms in Flasks

**Figure 4:**
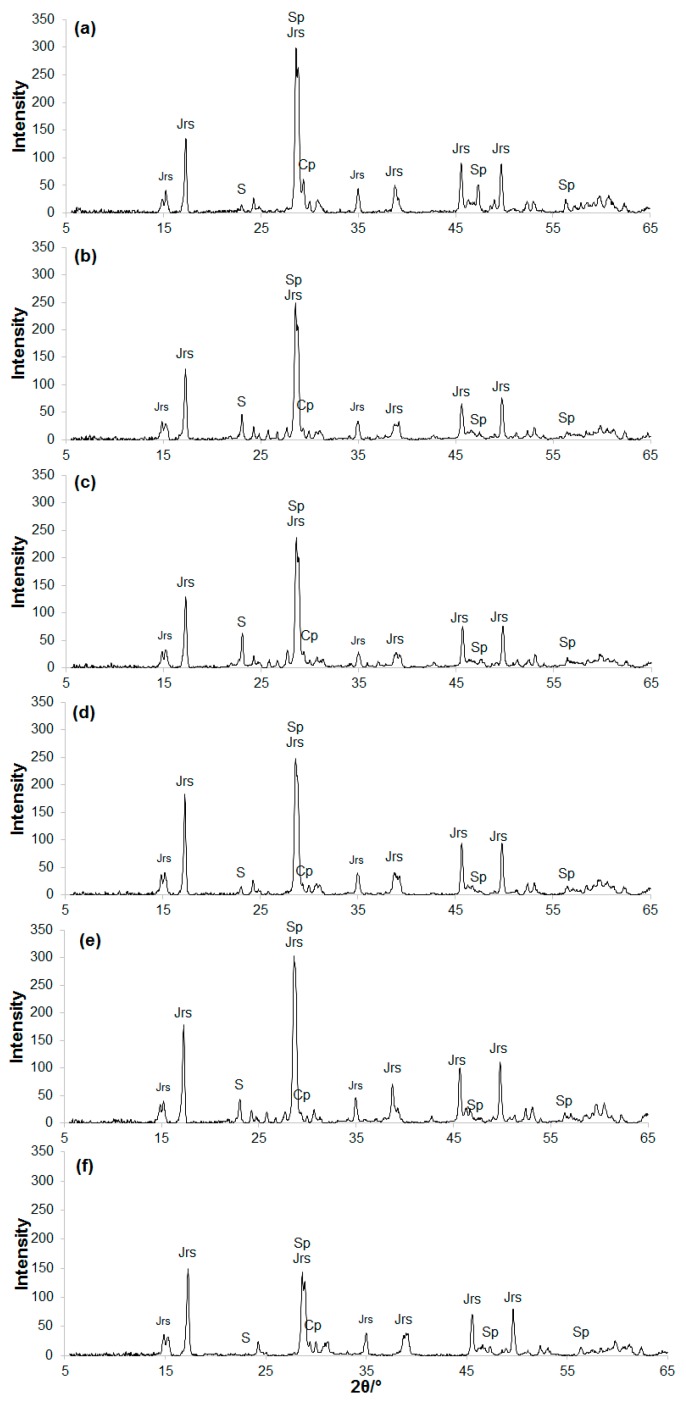
X-ray diffraction patterns of the residues of biooxidation by the following microorganisms: *A. caldus* MP3 (**a**), *L. ferriphilum* MP1 (**b**), *A. caldus* MP3 *+ L. ferriphilum* MP1 (**c**), *S. thermosulfidooxidans* MP2 (**d**), *F. acidiphilum* Y^T^ (**e**), mixture of all cultures (**f**) (Sp, sphalerite; Cp, chalcopyrite; Jrs, jarosite; S, elemental sulfur).

This study also aimed at understanding the roles of each predominant member of the acidophilic microbial community involved in the biooxidation of the products of ferric leaching of the zinc concentrate. Experiments with different pure cultures (strains MP1, MP2, and MP3) isolated from the bioreactor were carried out in flasks for 21 days. The liquid phase composition was the same as in the bioreactor, and the pulp density was 1% (*w*/*v*). As mentioned above, *F. acidiphilum* was identified as one of the prevalent microorganisms (according to the data on the metabarcoding analysis), but was not isolated into pure culture. The type strain of this species, *F. acidiphilum* Y^T^, which was included in the initial consortium that was used for leaching of the zinc sulfide concentrate, was, therefore, used in the experiments. The pH values changed within 1.5–2.0 in experiments with *A. caldus*. In the variants with *L. ferriphilum*, *S. thermosulfidooxidans*, and *F. acidiphilum*, the pH range was 1.8–2.0. No addition of calcium carbonate or sulfuric acid was required (due to a lower pulp density in flasks than in the bioreactor). Table 5 and Table 6 show the composition of the bioleach residues obtained with various microorganisms, as well as the oxidation levels of sulfide minerals and elemental sulfur. Figure 4a–f shows X-ray diffraction patterns of the mineralogical composition of these residues. In all variants, complete oxidation of pyrrhotite, which is the most readily oxidized sulfide mineral, was observed. *L. ferriphilum* MP1 and *F. acidiphilum* Y^T^ did not oxidize elemental sulfur, which agrees with their physiological characteristics. However, among all pure cultures, the highest oxidation levels of sulfide minerals were reached in experiments with *F. acidiphilum* Y^T^: 75 wt.% for sphalerite and 11 wt.% for chalcopyrite. Typically, strain Y^T^ oxidizes ferrous iron and pyrite [27,48], while oxidation of sulfide minerals, such as sphalerite, chalcopyrite, galenite, and antimonite, was not reported. To our knowledge, no studies showing efficient sphalerite oxidation by pure *Ferroplasma* cultures oxidizing ferrous iron were previously reported.

*L. ferriphilum* MP1 did not oxidize chalcopyrite and oxidized sphalerite at low efficiency. The highest level of elemental sulfur oxidation was reached when *A. caldus* was used. At the same time, no chalcopyrite oxidation was observed and insignificant sphalerite oxidation was probably due to the presence of ferric iron in the medium and dissolution with sulfuric acid. A comparison of *S. thermosulfidooxidans*, which is able to oxidize iron and sulfur, with the mixed culture consisting of the sulfur-oxidizing strain *A. caldus* MP3 and iron-oxidizing *L. ferriphilum* MP1 resulted in the same efficiency of sphalerite and chalcopyrite oxidation, while the level of sulfur oxidation was two times higher in the latter case. Moreover, although the mixed culture of *A. caldus* and *L. ferriphilum* oxidized sulfide minerals better than the pure cultures, the oxidation level of elemental sulfur by this mixture was more than four times lower than that of the pure culture of *A. caldus*. Probably, this was caused by the formation of additional amounts of elemental sulfur due to a higher level of oxidation of sphalerite and chalcopyrite by the combined culture. Moreover, inhibition of *A. caldus* in the presence of *L. ferriphilum* due to greater sensitivity of the sulfur-oxidizer to ferric iron than to ferrous iron was previously shown [49].

The mixture of all studied microorganisms showed the highest efficiency of oxidation of both sulfide minerals and elemental sulfur. This agrees with the results of numerous studies that reported the advantage of combined cultures of iron- and sulfur-oxidizing acidophiles over their pure cultures during oxidation of various substrates, including those containing chalcopyrite, pyrite, and covellite [50,51,52,53,54,55]. The most common explanation for the high efficiency of the microbial community is a more successful removal of the products of oxidation reactions (including elemental sulfur) from the surface of sulfide minerals. Microorganisms that can use organic substances for constructive or energy metabolism can play a role in the detoxification of the environment for autotrophic microorganisms. Toxicity of the microbial lysis products for *L. ferriphilum* and *A. ferrooxidans* was shown and it was more pronounced for the latter [56]. *Acidiphilum acidophilum* was able to increase the efficiency of metal bioleaching due to the utilization of organic substances, therefore decreasing the toxicity of the culture medium for autotrophic acidophiles [57]. A similar pattern of synergistic interaction between *F. acidiphilum* and *L. ferriphilum* was also observed [58]. The growth of chemomixotrophic *F. acidiphilum* was favored by the supernatant of chemolithoautotrophic *L. ferriphilum*, containing additional sources of the organic carbon (EPS and lysed cells) and vice versa, probably due to detoxification of the medium from organic compounds by mixotrophic *F. acidiphilum* and extracellular proteins of its “secretome” [58]. The study of the microbial community dynamics in various industrial processes of tank or heap biooxidation showed the dominance of the *Ferroplasmaceae* family members [59,60,61]. According to the results of our study, *F. acidiphilum* played a crucial role in the oxidation of sulfide minerals of the leach residue of the zinc concentrate by the mixture of microbial cultures. *A. caldus* played a key role in the oxidation of elemental sulfur. Probably, the efficient oxidation of elemental sulfur during the process of biooxidation was possible due to the gradual adaptation of *A. caldus* to ferric iron in the medium. After 21 days, the total cell abundance in the microbial community continued to increase due to the proceeding growth of the numerically predominant strain *A. caldus* MP3, while the pH value continued to decrease, indicating active sulfur oxidation.

**Figure 5:**
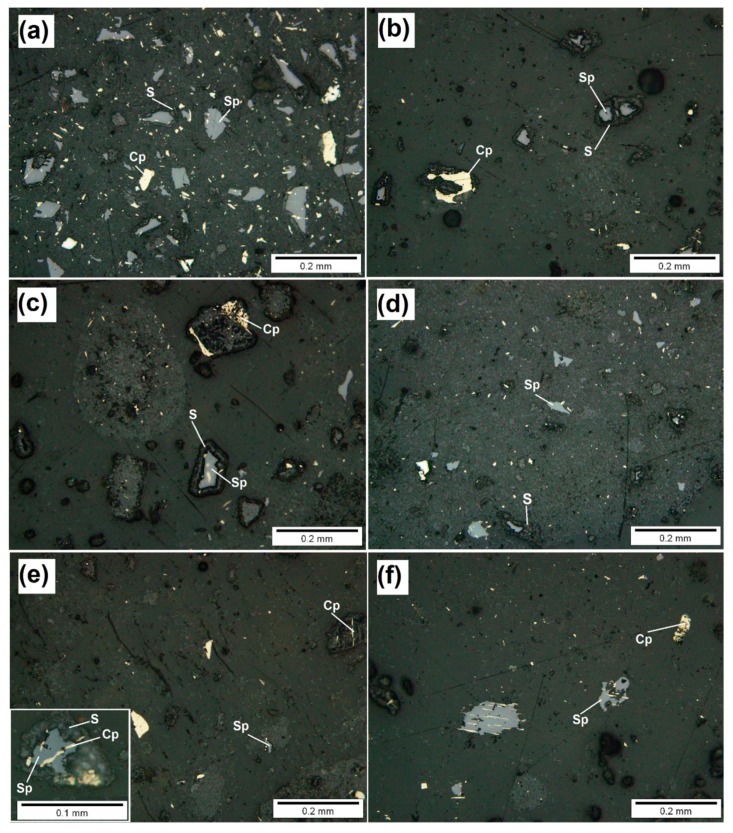
Micrographs of polished sections of the residues of biooxidation by the following microorganisms: *A. caldus* (**a**), *L. ferriphilum* (**b**), *A. caldus + L. ferriphilum* (**c**), *S. thermosulfidooxidans* (**d**), *F. acidiphilum* Y^T^ (**e**), mixture of all cultures (**f**) (Sp, sphalerite; Cp, chalcopyrite; S, elemental sulfur).

Figure 5a–f shows the micrographs of polished sections of the residues of biooxidation by different microorganisms. These images agree with the data on mineralogical and chemical analyses. The difference in the thickness of the sulfur layers on the surface of sulfides (mainly sphalerite) is of particular interest. After biooxidation, the “cleanest” surface of the minerals was observed when the mixture of all cultures was used (Figure 5f). Interestingly, unusual layers of reaction products surrounding the surface of sulfides (mainly sphalerite) were found in the variants with *L. ferriphilum* MP1 and the mixture of *L. ferriphilum* MP1 and *A. caldus* MP3 (Figure 5b,c). Two compact layers of sulfur and a thick porous sulfur layer between them formed a “capsule” surrounding the particles of sphalerite, which is the main source of reduced sulfur compounds during biooxidation. Somewhat similar sulfur layers of different porosity around sphalerite grains were observed during sphalerite biooxidation by *A. ferrooxidans* [62]. At the same time, it should be noted that jarosite was not formed on the surface of sphalerite [63].

Curiously, these well-defined capsules were formed only in the variants with *L. ferriphilum*. In the case of *F. acidiphilum*, sulfur layers had porous structure (Figure 4e) and did not impede sphalerite oxidation (Table 6). Some particles of mineral sulfides of the original ferric leach residue used for subsequent biooxidation were also partly covered with sulfur layers (Figure 3b). However, these layers did not form well-defined capsules as in the case of *L. ferriphilum*. Similarly, sphalerite particles were not completely covered with sulfur passivation layers when the pure culture of *A. caldus* MP3 was used (Figure 5a), which agrees with the data on highly efficient sulfur oxidation by this culture (Table 6).

According to these observations, we suggest that the main role in the formation of the capsule surrounding sphalerite particles belonged to *L. ferriphilum*. In addition to sulfur, organic extracellular polymeric substances (EPS) could also contribute to the formation of these layers. Thus, a recent study by Vardanyan et al. [64] indicated that *L. ferriphilum* cells excreted large amounts of EPS when grown either on soluble ferrous iron or solid pyrite. In the presence or absence of *A. thiooxidans*, *L. ferriphilum* cells were shown to form a pronounced EPS layer on the surface of sulfide minerals and secrete more polysaccharides into this layer during bioleaching [65]. At the same time, polysaccharides can complex Fe^3+^, which is beneficial for accumulating large amounts of Fe^3+^ between the EPS and mineral surface, thus promoting Fe^3+^ to attack and dissolve minerals [65]. Moreover, the presence of additional organic substrates in the culture medium of *Leptospirillum* spp. improved the rate and degree of attachment to a polymetallic mineral [66]. In our experiments, increased EPS synthesis by *L. ferriphilum* MP1 could also be due to the presence of yeast extract.

In general, such a pattern of sulfur passivation decreased sulfur availability for oxidation by *A. caldus* MP3 and prevented active mineral dissolution by *L. ferriphilum* MP1. As mentioned above, low sulfur oxidation by *A. caldus* in the mixture with *L. ferriphilum* can also be explained by partial inhibition of *A. caldus* activity by ferric iron [49]. The nature and functions of sulfur capsules observed in this study require further investigation and are of particular interest for the bioleaching/biooxidation of zinc concentrates.

Many studies reported a decrease in the rate of sphalerite oxidation or even its cessation due to the formation of sulfur layers acting as a diffusion barrier on the mineral surface [67] or due to the accumulation of jarosite [68]. Despite this fact, although in the case of *F. acidiphilum* Y^T^, which cannot oxidize sulfur, the biooxidation residue contained large amounts of both elemental sulfur and jarosite, it was characterized by the highest level of sphalerite oxidation. We, therefore, conclude that sphalerite oxidation is determined not only by the amount of reaction products on the surface of the mineral or in the biooxidation residue, but also by their qualitative characteristics, such as porosity, adhesive ability, and others, which may be associated with the species and strain composition.

## 4. Conclusions

Biooxidation of the ferric leach products (with the ferric iron solution obtained using an autotrophic microbial consortium) of the zinc sulfide concentrate by a microbial community for 21 days at 40 °C was studied. The solid leach product (leach residue) contained 29.7% of elemental sulfur. It was shown that:

- the final oxidation levels of sphalerite and chalcopyrite were 99 and 69%, respectively (compared to the original sulfide concentrate), which indicates the effectiveness of a two-step process for processing low-grade zinc concentrates;
- the biooxidation residue was mainly composed of jarosite and gypsum and contained 0.52% of zinc, 0.55% of copper, and 0.40% of elemental sulfur; therefore, it can be considered dump waste that is inert under aerobic conditions;
- the developed microbial community consisted of a sulfur-oxidizing bacterium *A. caldus*, an iron-oxidizing lithoautotroph *L. ferriphilum*, an iron-oxidizing mixotroph *F. acidiphilum*, iron- and sulfur-oxidizing mixotrophs *S. thermotolerans* and *S. thermosulfidooxidans*, as well as a heteroorganotroph *Cuniculiplasma* sp.;
- pure cultures of *A. caldus*, *L. ferriphilum*, and *S. thermosulfidooxidans*, isolated from the acidophilic microbial community, as well as *F. acidiphilum* Y^T^, oxidized sulfide minerals and elemental sulfur of the leach residue at lower efficiency than a mixture of these microorganisms;
- a mixture of *A. caldus* and *L. ferriphilum* oxidized elemental sulfur in the leach residue at a lower level than a pure culture of *A. caldus*;
- the crucial roles in the oxidation of sulfide minerals and sulfur of the ferric leach residue belonged to *F. acidiphilum* and *A. caldus*, respectively;
- sphalerite oxidation is determined not only by the amount of reaction products on the surface of the mineral or in the biooxidation residue, but also by their qualitative characteristics associated with the species and strain composition.

## Figures and Tables

**Table 1:** Contents of the main elements and minerals in the zinc concentrate and the leach residue.

	Content (wt.%)	
	Zinc Concentrate	Leach Residue
*Elements*		
Zn	43.3	6.17
Cu	2.03	1.82
Fe	13.6	17.3
S_SO4_^a^	1.38	6.0
S^0 a^	0	29.7
S_s_^a^	30.5	7.3
Ca	<0.1	<0.1
*Minerals*		
Sphalerite (ZnS)	65	9
Chalcopyrite (CuFeS_2_)	6	5
Pyrrhotite (Fe_1-x_S)	18	<1
Jarosite (XFe_3_(SO_4_)_2_(OH)_6_) ^b^	0	55

^a^ S^0^, elemental sulfur; S_S_, sulfur of sulfides; S_SO4_, sulfur of sulfates. ^b^ X = K, NH_4_, H_3_O^+^.

**Table 2:** The contents of elements and minerals in the solid phase after biooxidation of the products of ferric leaching of zinc concentrate by the microbial community in the bioreactor.

Yield of Solid Phase (wt.%)	Element Content (wt.%)					Mineral Content (wt.%)				
	Zn	Cu	Fe	S^0^	Ca	Sphalerite	Chalcopyrite	Pyrrhotite	Jarosite	Gypsum
212	0.52	0.55	6.90	0.40	18.3	<1	1.5	0	18	79

**Table 3:** The oxidation level (wt.%) of minerals and elemental sulfur in the solid phase after biooxidation of the products of ferric leaching of the zinc concentrate by the microbial community.

During Biooxidation				Total (After Ferric Leaching and Biooxidation)		
Sphalerite	Chalcopyrite	Pyrrhotite	Elemental Sulfur	Sphalerite	Chalcopyrite	Pyrrhotite
82	36	100	97	99	69	100

**Table 4:** The composition of the microbial community at the end of biooxidation in the bioreactor.

Cell Abundance Determined by Tenfold Dilutions (Species, Cells/mL)	The Proportion of the 16S rRNA Sequences in the Community (Genus, %)
*A. caldus*, 10^10^ *L. ferriphillum*, 10^5^ *Sulfobacillus thermosulfidooxidans*, 10^8^	*Acidithiobacillus*, 67 *Leptospirillum*, 0.75 *Sulfobacillus*, 8 *Ferroplasma*, 23 *Cuniculiplasma*, 0.75

**Table 5:** The contents of minerals and elemental sulfur in the solid phase during biooxidation of the products of ferric leaching of the zinc concentrate by various microbial cultures.

Microbial Culture	Content (wt.%)			
	S^0^	Sphalerite	Chalcopyrite	Jarosite
*A. caldus*	6.7	13	8	70
*L. ferriphilum*	20.0	5	3.5	69
*A. caldus* + *L. ferriphilum*	15.5	3.5	3	74
*S. thermosulfidooxidans*	9.2	3.5	3	78
*F. acidiphilum* Y^T^	13.3	1	2	76
Mixture of cultures	1.0	2	5	84

**Table 6:** The oxidation levels of minerals and elemental sulfur in the solid phase during biooxidation of the products of ferric leaching of the zinc concentrate by various microbial cultures.

Microbial Culture	Oxidation Level (wt.%)		
	S^0^	Sphalerite	Chalcopyrite
*A. caldus*	85	3	0
*L. ferriphilum*	0	17	0
*A. caldus* + *L. ferriphilum*	20	41	8
*S. thermosulfidooxidans*	54	42	11
*F. acidiphilum* Y^T^	0	75	11
Mixture of cultures	97	80	11
